# The role of surgery in older patients with T1-2N0M0 small cell lung cancer: A propensity score matching analysis

**DOI:** 10.3389/fonc.2022.958187

**Published:** 2022-09-30

**Authors:** Jing Ning, Tao Ge, Shuncang Zhu, Yingli Han, Suhong Ruan, Yuchen Ma, Rentao Liu

**Affiliations:** ^1^ Department of Oncology, The Affiliated Suzhou Hospital of Nanjing Medical University, Suzhou Municipal Hospital, Suzhou, China; ^2^ Department of Thoracic Surgery, Shanghai Pulmonary Hospital, Tongji University School of Medicine, Shanghai, China

**Keywords:** small cell lung cancer, older patients, prognosis, propensity score matching, surgery

## Abstract

**Background:**

Surgical resection could improve the survival of patients with early-stage small cell lung cancer (SCLC). However, there is a lack of dedicated studies concentrating on surgical treatment in older patients with T1-2N0M0 SCLC. Thus, we performed this population-based study to investigate whether older patients with T1-2N0M0 SCLC could benefit from surgery.

**Methods:**

We collected the data of patients with SCLC between 2000 and 2015 from the Surveillance, Epidemiology, and End Results Program database. Older patients (≥ 65 years) with T1-2N0M0 SCLC were included, and we converted the staging information into those of the eighth edition. The propensity score matching (PSM) was used to balance the distribution of clinical characteristics between surgery and no-surgery groups.

**Results:**

Before PSM, the distribution proportions of clinical characteristics in 1,229 patients were unbalanced. The Kaplan–Meier curves of overall survival (OS) and cancer-specific survival (CSS) showed that the patients in the surgery group were better than those in the non-surgery group (all *P* < 0.001). After 1:2 PSM, the distribution proportions of clinical characteristics in 683 patients were balanced (all *P* > 0.05). The OS and CSS of patients in the surgery group were still better than that of patients in the no-surgery group (all *P* < 0.001), and subgroup analysis showed that the surgery was a protective factor for OS and CSS in all clinical characteristics subgroups (almost *P* < 0.001). The multivariate Cox analysis further confirmed this result (OS: HR, 0.33; 95% CI, 0.27–0.39; *P* < 0.001; CSS: HR, 0.29; 95% CI, 0.23–0.36; *P* < 0.001). The result of subgroup analysis based on age, T stage, and adjuvant therapy showed that surgery was related to better OS and CSS compared with non-surgery group (almost *P* < 0.001) and that lobectomy exhibited the longer survival than sublobectomy. Age, sex, and race were the independent prognostic factors for OS in patients undergoing surgery, whereas only the factor of age affects the CSS in patients with surgery.

**Conclusions:**

Older patients with T1-2N0M0 SCLC can benefit significantly from surgical treatment, and lobectomy provides better prognosis than sublobectomy.

## Introduction

Lung cancer is the leading cause of malignancy incidence and mortality (1). Small cell lung cancer (SCLC) accounts for approximately 15% of total lung cancer cases and is characterized by rapid growth, high vascularity, early metastatic spread, significant sensitivity to chemotherapy and radiotherapy, and development of drug resistance during the course of disease, with a 5-year survival rate of 7% (2, 3). Thus, stage T1-2N0M0 SCLC only accounts for nearly 5% of patients diagnosed with SCLC, which have a better prognosis, with a 5-year survival up to 50% (4, 5). As the aged population increases, the diagnosis of cancer will continue to rise in older patients. Lung cancer has become a disease of the elderly, with the average age at diagnosis of 70 years (6, 7). The standard treatment for SCLC is chemotherapy alone or in combination with concurrent radiotherapy (8), but the rate of local recurrence is up to 50% in limited stage, although SCLC is sensitive to chemotherapy and radiotherapy (9, 10). Currently, the National Comprehensive Cancer Network (NCCN) guidelines recommended surgery for patients with clinical T1-2N0M0 SCLC (11). Moreover, some retrospective studies stated that patients with limited SCLC who underwent surgery had an excellent survival and 5-year survival rate of approximately 50% (5, 12). In addition, the previous study in terms of surgical approach showed that the survival of the lobectomy group was better than that of the wedge resection in patients with stage I-IIA SCLC (13, 14).

However, compared with young people, the older patients may be frail with complex underlying diseases, poor performance status, and increased treatment-related complications (15). Therefore, identifying the optimal treatment for older patients with early-stage SCLC is challenging. Previous research showed that comorbidity alone was not the reason to withhold standard therapy in limited SCLC (16). Because of the low enrollment of older patients in cancer randomized clinical trials (RCTs) (17), there was also a lack of evidence-based RCTs that surgery is superior to conservative management in terms of long-term survival benefits in the older population. Meanwhile, surgery and postoperative adjuvant therapy were significantly underused among the older population with early-stage SCLC (4, 13, 18). Consequently, there was still no consensus on whether the older patients (≥ 65 years) with T1-2N0M0 SCLC could benefit from surgery currently.

In this study, we performed strict matching of clinical data between the surgical and non-surgical groups by propensity score matching (PSM), so as to eliminate the confounding effect of clinical characteristics of the two groups. Finally, we evaluated the effect of surgery on the long-term survival in older patients (≥ 65 years) with T1-2N0M0 SCLC based on the Surveillance, Epidemiology, and End Results (SEER) Program database.

## Methods

### Patient selection

**Figure 1:**
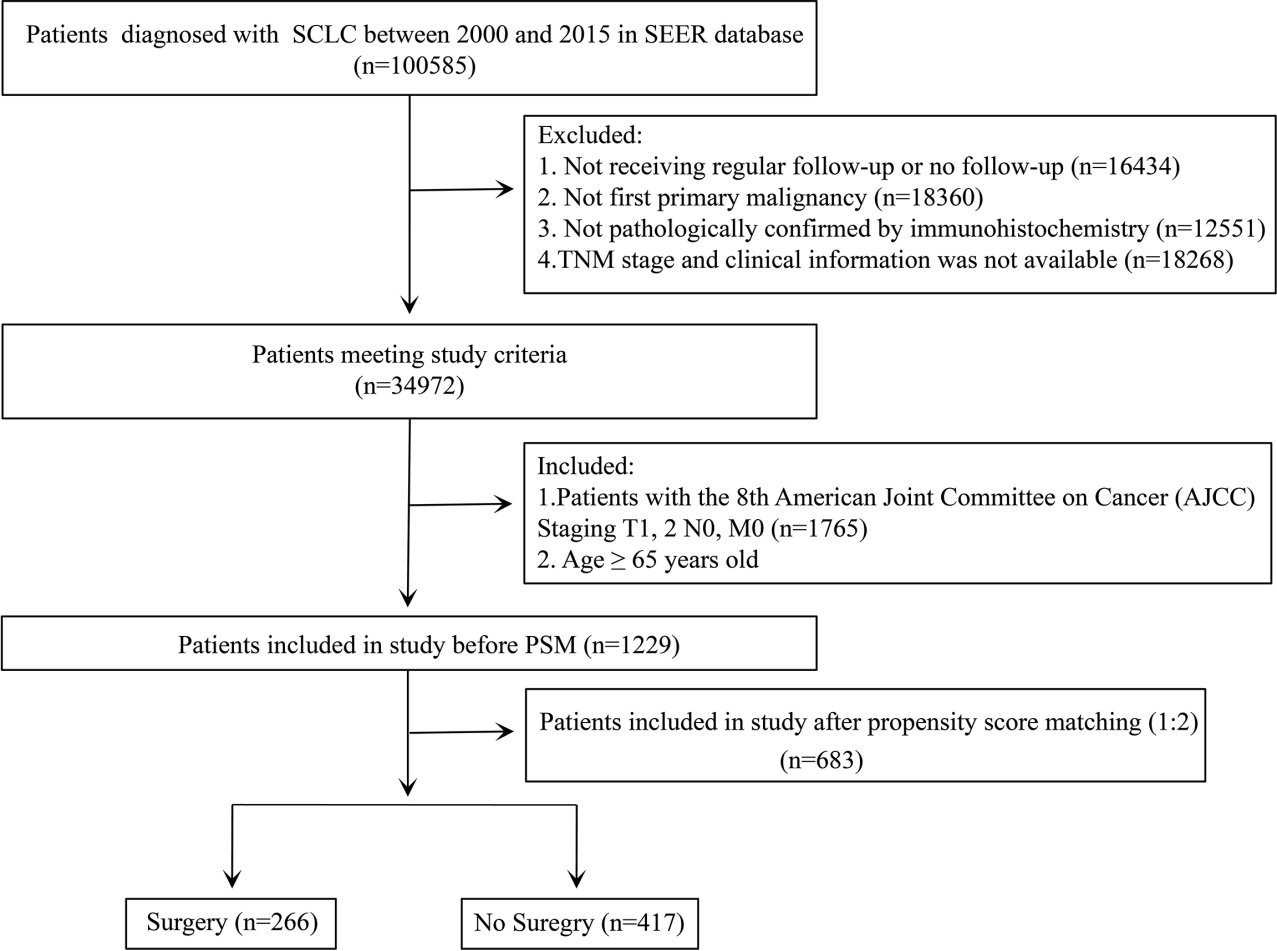
Flowchart for data filtration of older patients with T1-2N0M0 small cell lung cancer (SCLC).

The SEER database is an authoritative source for cancer statistics that covers approximately 28% of the US population and contains data on cancer occurrences in 18 areas of the United States. The selected patients diagnosed with SCLC were identified using the SEER * Stat version 8.3.9 (National Cancer Institute, Bethesda, MD, USA). The study cohort consisted of the patients with the International Classification of Disease for Oncology Third Edition (ICD-O-3) morphology codes (8041/3, 8042/3, 8043/3, 8044/3, and 8045/3) and site codes (C34.0, C34.1, C34.2, C34.3, C34.8, and C34.9). The exclusion criteria were as follows: (I) not receiving regular follow-up or no follow-up; (II) patients having at least one prior malignancy; (III) not pathologically confirmed by immunohistochemistry; and (IV) patients with missing information concerning primary tumor size (T), regional lymph node (N), or distant metastasis (M) stage and clinical information. After that, we also set up the including criteria for the patients meeting the above exclusion criteria: aged ≥ 65 years; patients with the eighth edition of American Joint Committee on Cancer (AJCC) staging system, stage T1-2N0M0 (**Figure 1**).

### Variables

To facilitate data analysis, we converted continuous variables into categorical variables. The extracted clinical information included sex, age (65–70, 71–80, and >80 years), race, laterality (left and right), T stage (T1a, T1b, T1c, and T2), surgery (surgery and no surgery), radiotherapy or not, chemotherapy or not, survival months, causes of death, and vital status. In terms of surgery, we defined the resection of less than one lobe as sublobectomy as some surgical procedures were not clear in the SEER database or the number was so small that we cannot analyze them separately. In addition, we converted the TNM categories for each patient according to the Collaborative Staging Manual and Coding Instructions for the eighth edition of the AJCC staging system using tumor size and tumor CS extension. For chemotherapy or radiotherapy, we were unable to define neoadjuvant or adjuvant therapy due to the lack of sequence of the treatment. The primary outcome was defined as overall survival (OS) and cancer-specific survival (CSS). The time of the last follow-up was November 2020. OS was defined as the interval between cancer diagnosis and death resulting from any cause or the last follow-up for patients still alive. CSS was defined as the length of time from cancer diagnosis to death from SCLC.

### Statistical analysis

The baseline characteristics of patients in the surgery group and the no-surgery group are described using frequencies and percentage. PSM was performed between the two groups to reduce potential bias and possible confounding interference. The baseline demographic data for the two groups were compared using the Student’s t-test or χ^2^ test and the Fisher’s exact test before and after PSM as deemed appropriate. Kaplan–Meier survival curves were plotted to assess distinctions in prognosis by applying the log-rank test. We used Cox proportional hazards regression analyses with both univariate and multivariate Cox regression analyses. Moreover, the multivariate Cox proportional hazards regression models were also performed to assess the risk factors in subgroup analyses. In addition, the forest plot of hazard ratios was constructed from univariate and multivariate Cox regression analyses. All data analyses were performed using RStudio version 4.1.2 (RStudio, Boston, MA, USA). A two-sided *P*-value < 0.05 was deemed significant.

## Result

### Baseline clinical characteristics

A total of 1,229 patients aged ≥65 years who had been diagnosed with T1-2N0M0 SCLC were included in our study. Of the population included, 71.6% of patients (880 patients) did not receive the surgical resection. The baseline characteristics of patients and tumors are shown in **Table 1**. The result showed that the distribution frequencies of some characteristics, including age, race, T stage, radiotherapy, and chemotherapy, were quite unbalanced between the surgery group and the no-surgery group. The patients in the surgery group were fewer than that in the no-surgery group among different age groups. The no-surgery group was associated with the white race and the larger size of the tumor. In terms of therapy, the no-surgery group was more likely to have radiotherapy or chemotherapy. Given the unbalanced distribution of these factors between surgery and non-surgery groups, there is a need to reduce the interference from these factors to better determine the significance of surgery for prognosis in the older patients.

**Table 1:** The clinicopathologic characteristics of older patients with T1-2N0M0 SCLC before propensity score matching.

Characteristics	Total (N, %)	No Surgery	Surgery	P-value
All	1,229	880 (71.60)	349 (28.4)	
Age (year)				<0.001
>80	204 (16.60)	166 (18.86)	38 (10.89)	
65–70	416 (33.85)	272 (30.91)	144 (41.26)	
71–80	609 (49.55)	442 (50.23)	167 (47.85)	
Sex				0.908
Female	667 (54.27)	479 (54.43)	188 (53.87)	
Male	562 (45.73)	401 (45.57)	161 (46.13)	
Race				0.004
Black	94 (7.65)	79 (8.98)	15 (4.30)	
Other	50 (4.07)	41 (4.66)	9 (2.58)	
White	1085 (88.28)	760 (86.36)	325 (93.12)	
Laterality				0.434
Left	532 (43.29)	388 (44.09)	144 (41.26)	
Right	695 (56.55)	490 (55.68)	205 (58.74)	
Unknown	2 (0.16)	2 (0.23)	0 (0.00)	
T stage (eighth edition)				<0.001
T1a	54 (4.39)	18 (2.05)	36 (10.32)	
T1b	345 (28.07)	195 (22.16)	150 (42.98)	
T1c	376 (30.59)	280 (31.82)	96 (27.51)	
T2	454 (36.94)	387 (43.98)	67 (19.20)	
Radiotherapy				<0.001
No	648 (52.73)	363 (41.25)	285 (81.66)	
Yes	581 (47.27)	517 (58.75)	64 (18.34)	
Chemotherapy				<0.001
No	446 (36.29)	281 (31.93)	165 (47.28)	
Yes	783 (63.71)	599 (68.07)	184 (52.72)	

SCLC, small cell lung cancer.

Univariate Cox analysis showed that the OS of patients was associated with age, T stage, surgery, radiotherapy, and chemotherapy (**Figure S1A**). Further multivariate Cox analysis showed that aged 65–70 years, right laterality, surgery, radiotherapy, and chemotherapy were the positive prognostic factors for OS (**Figure S1C**). Analogously, the variables of age, T stage, surgery, radiotherapy, and chemotherapy were related to the CSS of patients through univariate Cox analysis (**Figure S1B**). Age, laterality, surgery, radiotherapy, and chemotherapy were the independent predictive factors for CSS (**Figure S1D**). The Kaplan–Meier curves showed that the OS and CSS of patients aged ≥65 years with T1-2N0M0 SCLC who underwent surgery were significantly better than those who did not undergo surgery (both P < 0.001; **Figure S2**).

### Survival analysis and multivariate Cox analysis after propensity score matching

After 1:2 PSM of seven clinical characteristics, a total of 683 patients were included in the analyses, which contain 417 patients in the non-surgery group and 266 patients in the surgery group. The distribution of these baseline characteristics was balanced between the two propensity-matched groups (both P > 0.05; **Table 2**).

**Table 2:** The clinicopathologic characteristics of older patients with T1-2N0M0 SCLC after propensity score matching.

Characteristics	Total (N,%)	No Surgery	Surgery	P-value
All	683	417	266	
Age (year)				0.175
>80	92 (13.47)	59 (14.15)	33 (12.41)	
65–70	233 (34.11)	131 (31.41)	102 (38.35)	
71–80	358 (52.42)	227 (54.44)	131 (49.25)	
Sex				0.399
Female	383 (56.08)	228 (54.68)	155 (58.27)	
Male	300 (43.92)	189 (45.32)	111 (41.73)	
Race				0.982
Black	31 (4.54)	19 (4.56)	12 (4.51)	
Other	22 (3.22)	13 (3.12)	9 (3.38)	
White	630 (92.24)	385 (92.33)	245 (92.11)	
Laterality				0.653
Left	291 (42.61)	181 (43.41)	110 (41.35)	
Right	392 (57.39)	236 (56.59)	156 (58.65)	
T stage (eighth edition)				0.366
T1a	26 (3.81)	15 (3.60)	11 (4.14)	
T1b	240 (35.14)	139 (33.33)	101 (37.97)	
T1c	220 (32.21)	133 (31.89)	87 (32.71)	
T2	197 (28.84)	130 (31.18)	67 (25.19)	
Radiotherapy				0.054
No	497 (72.77)	292 (70.02)	205 (77.07)	
Yes	186 (27.23)	125 (29.98)	61 (22.93)	
Chemotherapy				0.461
No	300 (43.92)	178 (42.69)	122 (45.86)	
Yes	383 (56.08)	239 (57.31)	144 (54.14)	

SCLC, small cell lung cancer.

**Figure 2:**
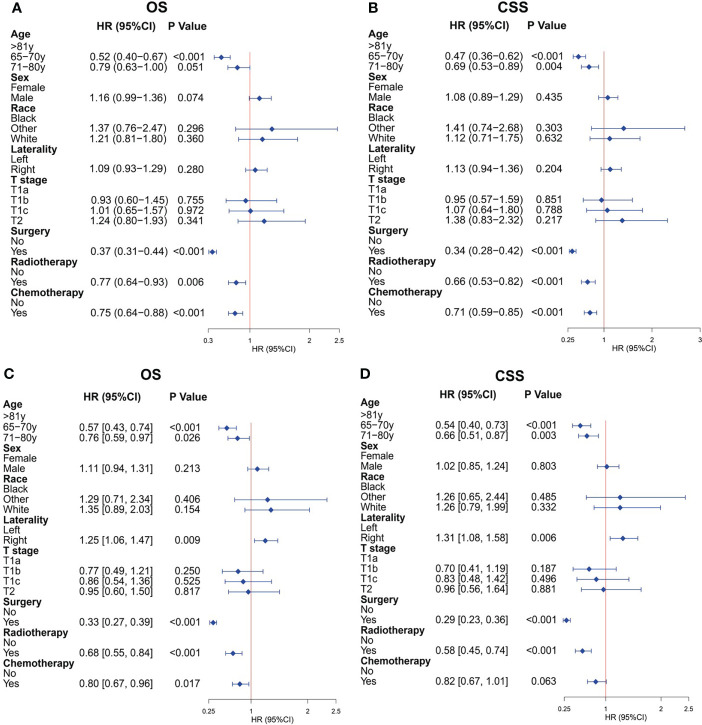
Cox regression analysis for overall survival (OS) and cancer-specific survival (CSS) of older patients with T1-2N0M0 small cell lung cancer (SCLC) after propensity score matching. **(A)** Univariate Cox analysis for OS. **(B)** Univariate Cox analysis for CSS. **(C)** Multivariate Cox analysis for OS. **(D)** Multivariate Cox analysis for CSS.

**Figure 3:**
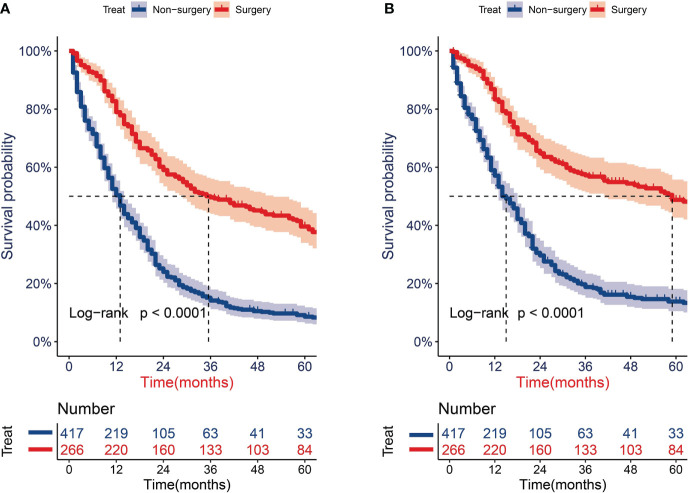
Survival analysis for overall survival (OS) and cancer-specific survival (CSS) of older patients with T1-2N0M0 small cell lung cancer (SCLC) after propensity score matching. **(A)** KM curves of OS. **(B)** KM curves of CSS.

After PSM, the univariate Cox analysis showed that aged 65–70 years, surgery, radiotherapy, and chemotherapy were related to the OS of patients (**Figure 2A**), whereas aged 65–70 and 71–80 years, surgery, radiotherapy, and chemotherapy were associated with the CSS of patients (**Figure 2B**). Through further multivariate Cox analysis, the result showed that aged 65–70 and 71–80 years, tumors located on the left side, surgery, radiotherapy, and chemotherapy were the positive predictive factors for OS (**Figure 2C**); aged 65–70 and 71–80 years, tumors located on the right side, surgery, and radiotherapy were the independent prognostic factors for CSS (**Figure 2D**). The Kaplan–Meier survival analysis after PSM showed that the OS of patients aged ≥65 years with T1-2N0M0 SCLC who underwent surgery were significantly better than those who did not undergo surgery (P < 0.001; **Figure 3A**). The median OS time of the surgery group was 35 months, which was significantly longer than the median OS time of the non-surgery group (13 months). After that, the median CSS time of the surgery group was also longer than that in the non-surgery group, with the median CSS time being 59 months in the surgery group and 14 months in the non-surgery group (P < 0.001; **Figure 3B**).

### Subgroup analysis of OS and CSS in subgroups of clinical characteristics

**Figure 4:**
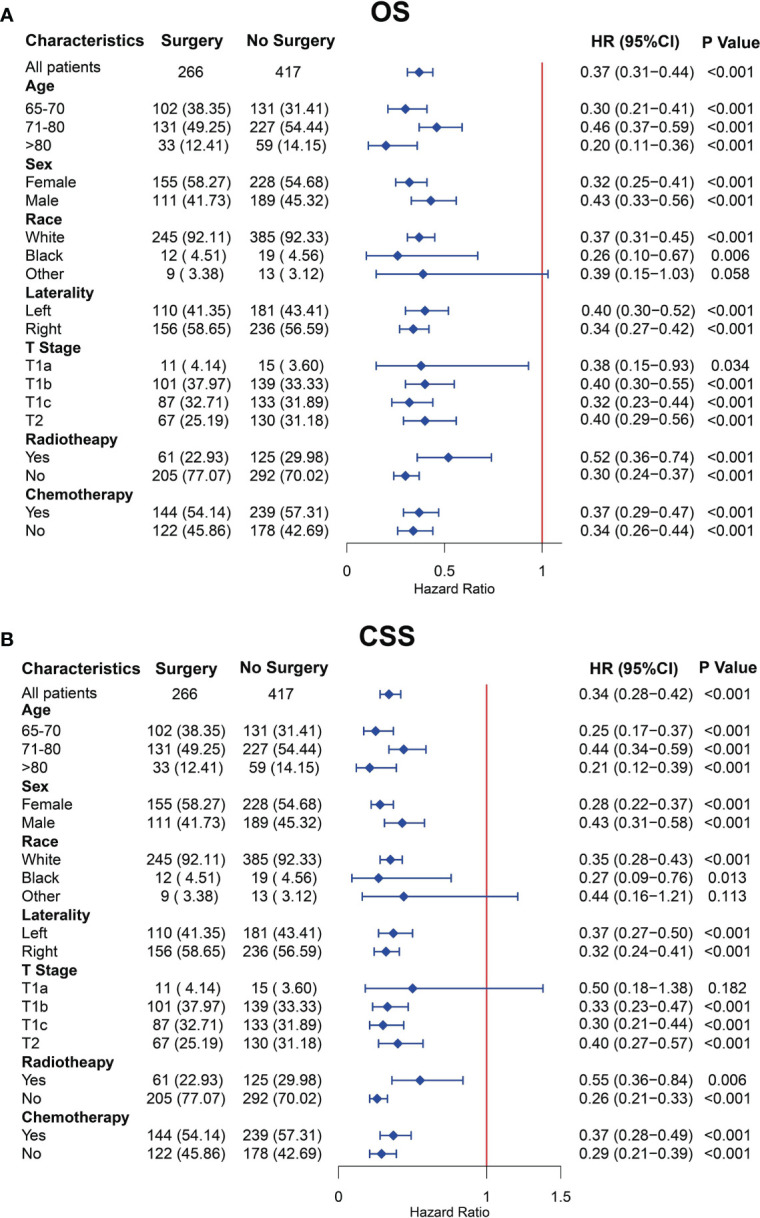
Subgroup analysis for overall survival (OS) **(A)** and cancer-specific survival (CSS) **(B)** of older patients with T1-2N0M0 small cell lung cancer (SCLC) after propensity score matching.

To better minimize the interference of other factors except for surgery on the prognosis and better determine the protective role of surgery on prognosis after PSM, we performed the subgroup analyses of all clinical characteristics. The OS subgroup analysis showed that the treatment of surgery was a protective factor for OS for almost clinical characteristics, except for the other subgroup of the race (**Figure 4A**). The CSS subgroup analysis showed that the surgical treatment was a protective factor for CSS for almost clinical characteristics, except for the other subgroup of race and the T1a subgroup of T stage (**Figure 4B**). The abovementioned clinical subgroups presented statistically insignificant differences in the OS or CSS between the surgery and no-surgery groups because the number of these subgroups was limited.

### Kaplan–Meier survival analysis of the OS and CSS for surgical treatment between different subgroups

**Figure 5:**
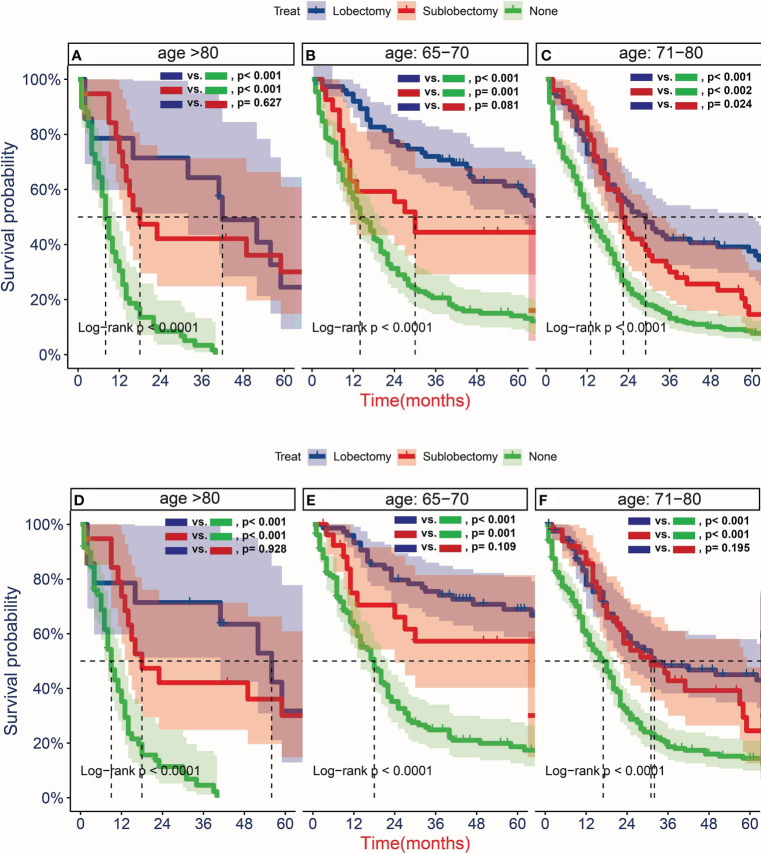
Kaplan–Meier estimates of overall survival (OS) **(A–C)** and cancer-specific survival (CSS) **(D–F)** for T1-2N0M0 patients with small cell lung cancer (SCLC) aged 65–70 years old **(B, E)**, 71–80 years old **(C, F)**, and >80 years old **(A, D)**stratified by surgery strategy after propensity score matching.

**Figure 6:**
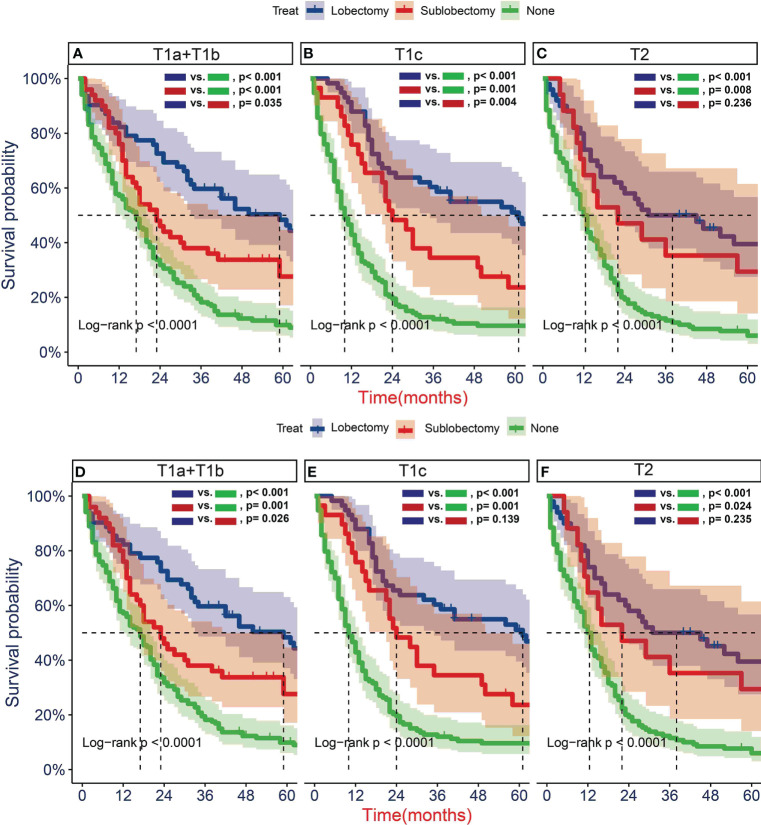
Kaplan–Meier estimates of overall survival (OS) **(A–C)** and cancer-specific survival (CSS) **(D–F)** for T1-2N0M0 patients with small cell lung cancer (SCLC) with stage T1a + T1b **(A, D)**, T1c **(B, E)**, T2 **(C, F)** stratified by surgery strategy after propensity score matching.

**Figure 7:**
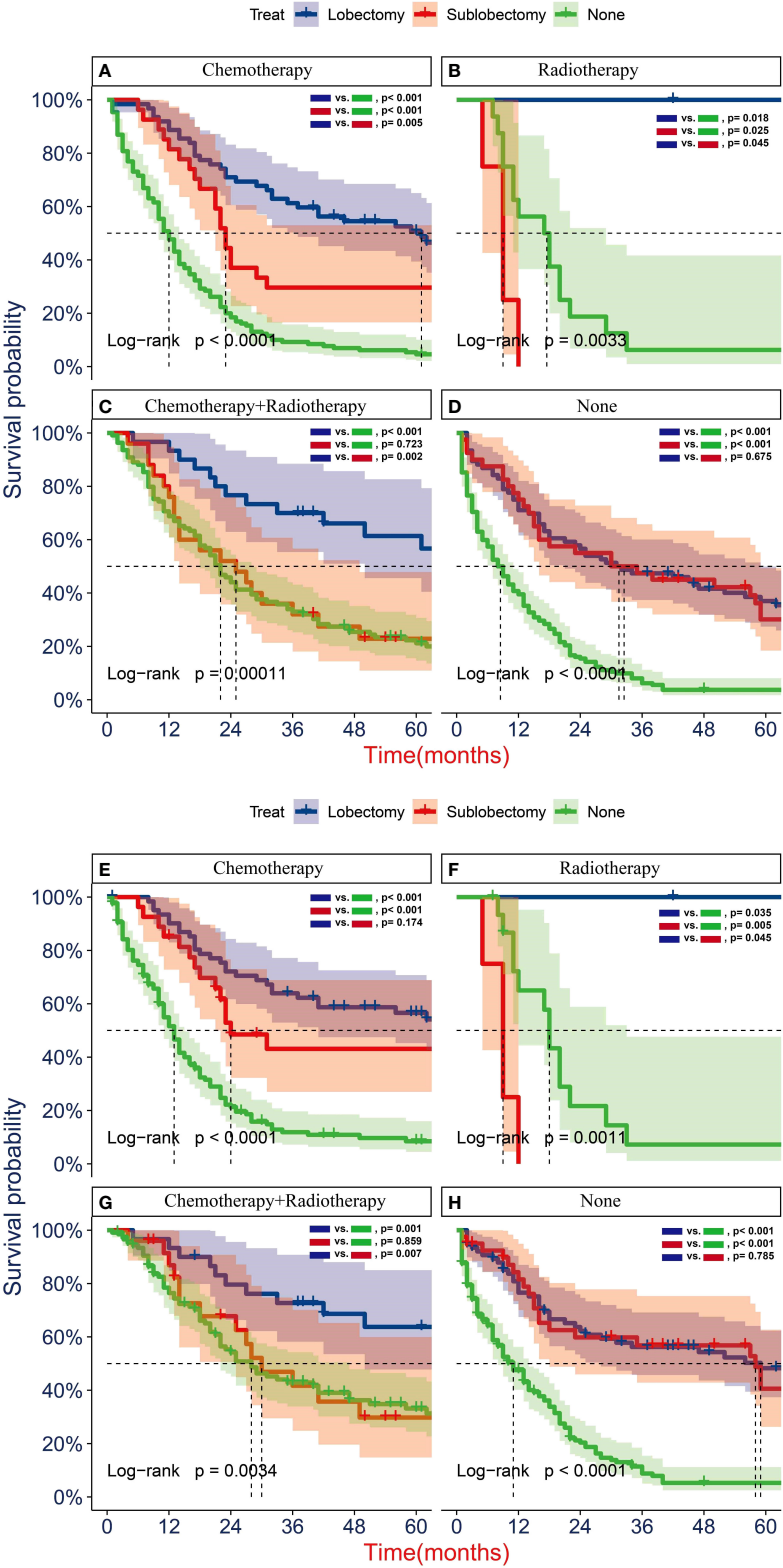
Kaplan–Meier analyses of overall survival (OS) **(A–D)** and cancer-specific survival (CSS) **(E–H)** for T1-2N0M0 patients with small cell lung cancer (SCLC) with chemotherapy **(A, E)**, radiotherapy **(B, F)**, radiotherapy plus chemotherapy **(C, G)**, and no radiotherapy or chemotherapy **(D, H)** stratified by surgery strategy after propensity score matching.

To further determine the protective effect of surgical procedure on OS and CSS, we performed the subgroup analysis in different age, tumor size, and treatment groups after PSM. The result showed that the surgery group had a better prognosis than the non-surgery group regardless of OS or CSS, but the difference between the two surgery strategies’ OS and CSS was not statistically significant in all age subgroups **Figures 5A, B, D–F**, except the OS in patients aged 71–80 years (**Figure 5C**, P = 0.024). However, all trends in survival benefits favored lobectomy over sublobectomy. In terms of the tumor size, we assembled the T1a and T1b as the group of T1a + T1b because the number of T1a group was limited with only 26 patients after PSM. The prognosis of two surgery strategies was better than that of no-surgery group in all T subgroups. The sublobectomy group had a worse prognosis than the lobectomy group in T1a + T1b stage subgroup regardless of OS or CSS (**Figures 6A, D**), whereas there was no significant difference in OS and CSS between the two different surgery strategies in the T2 subgroup (**Figures 6C, F**). In T1c subgroup analyses, the lobectomy group had a better prognosis than sublobectomy in OS, not in CSS, but the trend in survival benefit also favored lobectomy (**Figures 6B, E**). In terms of therapy, the surgery group all achieved better OS and CSS than the non-surgery group in patients who had chemotherapy alone (**Figures 7A, E**), radiotherapy alone (**Figures 7B, F**), and no chemotherapy or radiotherapy (**Figures 7D, H**), but the difference of prognosis in OS and CSS was insignificant between sublobectomy and no-surgery groups in patients who received chemotherapy plus radiotherapy (**Figures 7C, G**). For patients in the chemotherapy group, the lobectomy group could improve the prognosis in OS rather than CSS compared with the sublobectomy group, but the difference in OS and CSS of patients who received radiotherapy and chemotherapy plus radiotherapy was significant. However, this result was not well persuasive for the limited samples in the radiotherapy group. In no-chemotherapy or radiotherapy subgroup analyses, the OS and CSS of sublobectomy and lobectomy were comparable, but the outcomes were better than that of patients who did not undergo surgery (**Figures 7D, H**).

### Prognostic factors of patients in the surgery group

**Figure 8:**
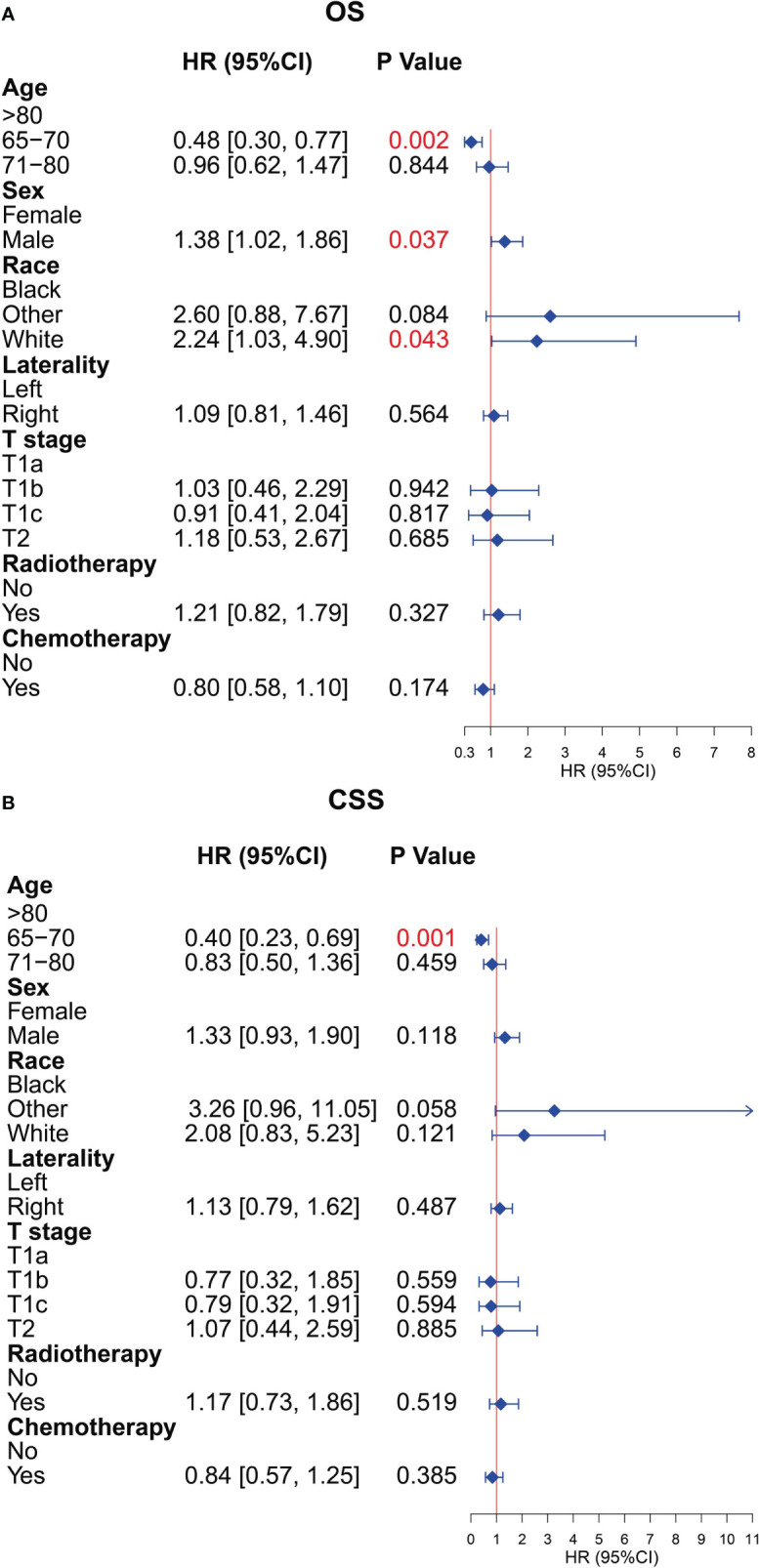
Multivariate Cox analysis for overall survival (OS) and cancer-specific survival (CSS) of the older patients with T1-2N0M0 small cell lung cancer (SCLC) who underwent surgery. **(A)** Multivariate Cox analysis for OS. **(B)** Multivariate Cox analysis for CSS.

To further explore the prognostic factors of older patients with T1-2N0M0 SCLC who underwent surgery, we performed the multivariate Cox analysis of the clinical characteristics of patients in the surgery group. The result presented that the characteristics of age (65–70 years), sex (female), and race (black) were the statistically positive influence factors for OS of patients (**Figure 8A**). After that, we find that just the factor of age (65–70 years) has a positive effect on the CSS of patients (**Figure 8B**).

## Discussion

The SEER database is currently the largest database of tumor clinical information in the world, which can help reduce the cancer burden among the US population. Many significant problems in clinical practice have been published using the SEER database in recent years (19, 20). However, the SEER database covers a long period and contains multiple different editions of the AJCC tumor staging system and other indicators, so it was so challenging to compare the results of the delivered research using the SEER database (21). Because of this reason, we converted the TNM staging of each patient into those of the eighth edition to guarantee that the study population information conformed to the current treatment guidelines. Notably, our study could provide credible and practical medical evidence for clinical decision-making of treatment in older patients with T1-2N0M0 SCLC through this approach.

The average age of patients diagnosed with SCLC increased, and the proportion of patients with SCLC older than 70 years had increased from 23% in 1975 to 44% in 2010 (22, 23). After that, the frequency of detecting early-stage lung cancer will be likely to increase as CT screening for lung cancer becomes more commonplace in recent years (24). Currently, the NCCN guidelines recommend surgery for selected cases of clinical stage T1-2N0M0 SCLC (25). However, considering the potential multiple comorbidities, increased treatment-related complications, decreased functional status, relatively high mortality in the older adult (26–29), and SCLC that is characterized by rapid growth and early metastasis, surgery is rarely performed in older patients even if their SCLC is at an early stage, and it was controversial whether the survival benefits of surgical treatment are significant for older patients. In our study, we also observed that the rate of surgery decreased with age increasing (34.6%, 27.4%, and 18.6% for the age subgroups 65–70, 71–80, and >80 years, respectively), and the surgical treatment could provide better prognosis than without surgery. Although there were several studies exploring the benefits of surgery in patients with early-stage SCLC, they did not stratify specifically by age and surgical procedure, and some of them had confounder interference (15, 30–32). Previous study also showed that age was the independent prognostic factor for patients with SCLC who received surgical treatment (33), which was consistent with the result of our research (**Figure 8**).

Before PSM, our results demonstrated that surgical treatment was the most significant protective factor of all clinical factors for OS and CSS, although a severe imbalance in the distribution of clinicopathological features between the surgery and non-surgery groups existed in our study. Whereas, the biases in data distribution in terms of baseline characteristics would interfere with the comparison between groups and the accuracy of the Cox regression model (34, 35). To determine the benefits of surgery in older patients and reduce confounding factor interference between the surgery group and the non-surgery group, we performed the 1:2 PSM to balance the distribution of a total of seven clinical characteristics so that the OS and CSS could be compared between the two groups at similar baselines and with a convincing result. After 1:2 PSM, with a total of 683 older patients with T1-2N0M0 SCLC, our results showed that the surgery remains the most important independent prognostic factor for older patients with T1-2N0M0 SCLC, and patients who underwent surgery achieved significantly better OS and CSS than those who did not undergo surgery (*P* < 0.001). Subgroup analysis also showed that surgical intervention was a protective factor for OS and CSS for almost clinical characteristics in older patients with SCLC. These results showed that a more aggressive treatment strategy may be beneficial in older patients with T1-2N0M0 SCLC, leading to a better survival of OS and CSS for these patients. Moreover, our study also noted that the factor of age 65–70 years was a protective factor of prognosis in older patients with SCLC undergoing surgery regardless of OS or CSS. Sex and race were independent predictors of OS in surgical patients and were not statistically significant in CSS, which is similar to the results of previous studies (15, 30, 32). Our study implied that these factors should be evaluated in detail before surgery and that intensive follow-up should be carried out for this special subset of patients although they have received surgical treatment.

The standard treatment for patients with limited-stage SCLC is chemotherapy and radiotherapy. However, considering the several physiological changes of organ function in older patients that could alter drug pharmacokinetics and have an impact on cytotoxic chemotherapy tolerability and toxicity, the treatment regimens may be different among different age (36). Ludbrook et al. analyzed retrospectively 174 patients with limited-stage SCLC and divided into three age groups: <65, 65–74, and ≥ 75 years. They found that increasing age was significantly associated with fewer diagnostic scans, less intensive chemotherapy regimens, fewer cycles, and lower total doses (37). In addition, there are some studies suggesting that the dose and frequency of radiotherapy were either less intensive in the elderly or comparable between younger and elderly patients (37, 38). After that, the local relapse occurs in up to 80% of limited-stage patients managed with chemotherapy alone, although SCLC was significantly sensitive to chemotherapy (39). Some data revealed that up to 16% of limited-stage SCLC died from a relapse confined to the thorax (40). Previous studies asserted that the treatment of operation could prevent local recurrence and improve survival in patients with SCLC (41, 42). A retrospective study published by Jin et al. also suggested that patients with T1-2N0 SCLC may benefit from surgery as local therapy, whereas patients with T3N0 or T1-2N1 SCLC may consider radiotherapy as local therapy (43). The American College of Chest Physicians and the American Society of Clinical Oncology also recommends surgery for patients with stage I SCLC, followed by adjuvant chemotherapy including platinum agent and etoposide (44, 45). In our study, we found that there was survival benefit for older patients who received surgery combined with chemotherapy or/plus radiotherapy compared with chemotherapy or/plus radiotherapy alone and that lobectomy may be the best choice, which was consistent with previous results (13, 42, 46). From the above results, it suggested that surgical treatment combined with adjuvant therapy may further improve the local control to prolong survival and supported the role of surgery in multimodality therapy for older patients with T1-2N0M0 SCLC.

According to the NCCN guidelines, surgery is recommended for patients with T1-2N0M0 SCLC, and it points out that lobectomy is superior to sublobectomy (25). However, many patients with early-stage SCLC also undergo sublobectomy for various reasons (47). To the best of our knowledge, few studies have discussed whether sublobectomy can achieve the same survival outcomes comparable to lobectomy in older patients (≥ 65 years) with T1-2N0M0 SCLC and which type of surgery combined with adjuvant therapy is most effective for OS and CSS currently. In our study, we find that the trend in OS and CSS benefits favored lobectomy over sublobectomy, and all achieved better survival than patients without surgery, although there was generally no statistical difference between the two surgical procedures in almost age subgroups. These results presented that sublobectomy could be considered in older patients with SCLC when patients cannot tolerate lobectomy due to various reasons like multiple comorbidities or poor pulmonary function. In terms of tumor size, our study found that lobectomy was the priority choice compared with sublobectomy for tumors with tumor size less than 5 cm, because lobectomy could have longer OS and CSS than sublobectomy. The above results were similar to those of previous studies (30, 48). Meanwhile, we also found that surgery combined with chemotherapy plus/or radiotherapy could achieve better survival than chemotherapy plus/or radiotherapy alone, which is consistent with the result of the previous report (13). Moreover, patients who underwent lobectomy continued to have better survival in our study. Moreover, in the non-treatment subgroup, we find that the sublobectomy seems to achieve the same therapeutic effect as lobectomy regardless of OS and CSS and that the surgery group had a better prognosis than the patients without any therapy, which represented that the patients who just received the treatment of surgery could also achieve survival benefit as older patients do. The occurrence of this phenomenon was possibly associated with the poorer performance status or relatively short life expectancy of this population. For the patients with chemotherapy plus radiotherapy, these patients may have the high risk of metastasis and recurrence due to the larger tumor size or special location of the tumor. We find that the OS and CSS of patients with sublobectomy were comparable with that of patients in the no-surgery group, which achieved worse prognosis than patients with lobectomy. Thus, this special subset of older patients still could benefit from aggressive surgical treatment regardless of OS or CSS, and lobectomy should be the prior choice in older patients.

The current study had some limitations. First, as a retrospective study, the population selection may be biased inevitably and could not control for confounding factors as strictly as prospective studies. Although we have performed the PSM to reduce the potential bias, there might be a potential unknown bias that the PSM failed to rectify. After that, it is not clear how patients were selected for different treatment in the SEER database. Second, the SEER database lacked routinely available data including performance status, lung function, smoking status, and comorbidities. In particular, comorbidities could greatly influence treatment strategies and prognosis assessment and might be the reason why patients who undergo surgery have better survival than those who did not. Third, the information on the status of surgical margin, disease-free survival, chemotherapy regimen and cycles, radiotherapy dose and location, and further treatment after recurrence was not available. Moreover, we are uncertain whether these factors had an impact on our study, for which we should draw the conclusions carefully. In addition, the data in our study were extracted from the American population, and the results need to be verified using the data from Chinese population. Overall, further multicenter prospective studies with relatively complete information of clinicopathological variables, performance status, and treatments in detail should be performed to validate our conclusions and provide more reliable clinical guidance.

In conclusion, our study found that the long-term survival of older patients with T1-2N0M0 SCLC who received surgical treatment was significantly better than that of patients who did not undergo surgery after balancing all clinical characteristics and that lobectomy could provide a better prognosis than sublobectomy. For patients unsuitable for lobectomy, this special subset of patients also could benefit from sublobectomy. Age, sex, and race were independent prognostic factors of survival outcomes in older patients undergoing surgery. Therefore, the surgery should be performed for older patients with T1-2N0M0 SCLC after careful consideration and assessment combined with relevant clinical factors, but further exploration in larger prospective clinical trials is also needed to validate our conclusions.

## Data availability statement

The raw data supporting the conclusions of this article will be made available by the authors, without undue reservation.

## Author contributions

All authors participated in manuscript writing and approved the final version of the manuscript. JN, TG, YM, SZ conceived and designed the analysis. Collection and assembly of data were performed by JN, TG, SZ, YH, SR. Analysis and interpretation of the data were supported by JN, TG, SZ. YM, RL conducted a critical review of the manuscript, contributing important intellectual content.

## Conflict of interest

The authors declare that the research was conducted in the absence of any commercial or financial relationships that could be construed as a potential conflict of interest.

## Publisher’s note

All claims expressed in this article are solely those of the authors and do not necessarily represent those of their affiliated organizations, or those of the publisher, the editors and the reviewers. Any product that may be evaluated in this article, or claim that may be made by its manufacturer, is not guaranteed or endorsed by the publisher.
