# Supplementary material for: The role of surgery in older patients with T1-2N0M0 small cell lung cancer: A propensity score matching analysis
Source: Front Oncol. 2022 Sep 30;12:958187. doi: 10.3389/fonc.2022.958187 (PMC9565197; doi:10.3389/fonc.2022.958187)
Supplement: Supplementary file 1 [file DataSheet_1.docx]

Supplementary Material

## Supplementary Figures


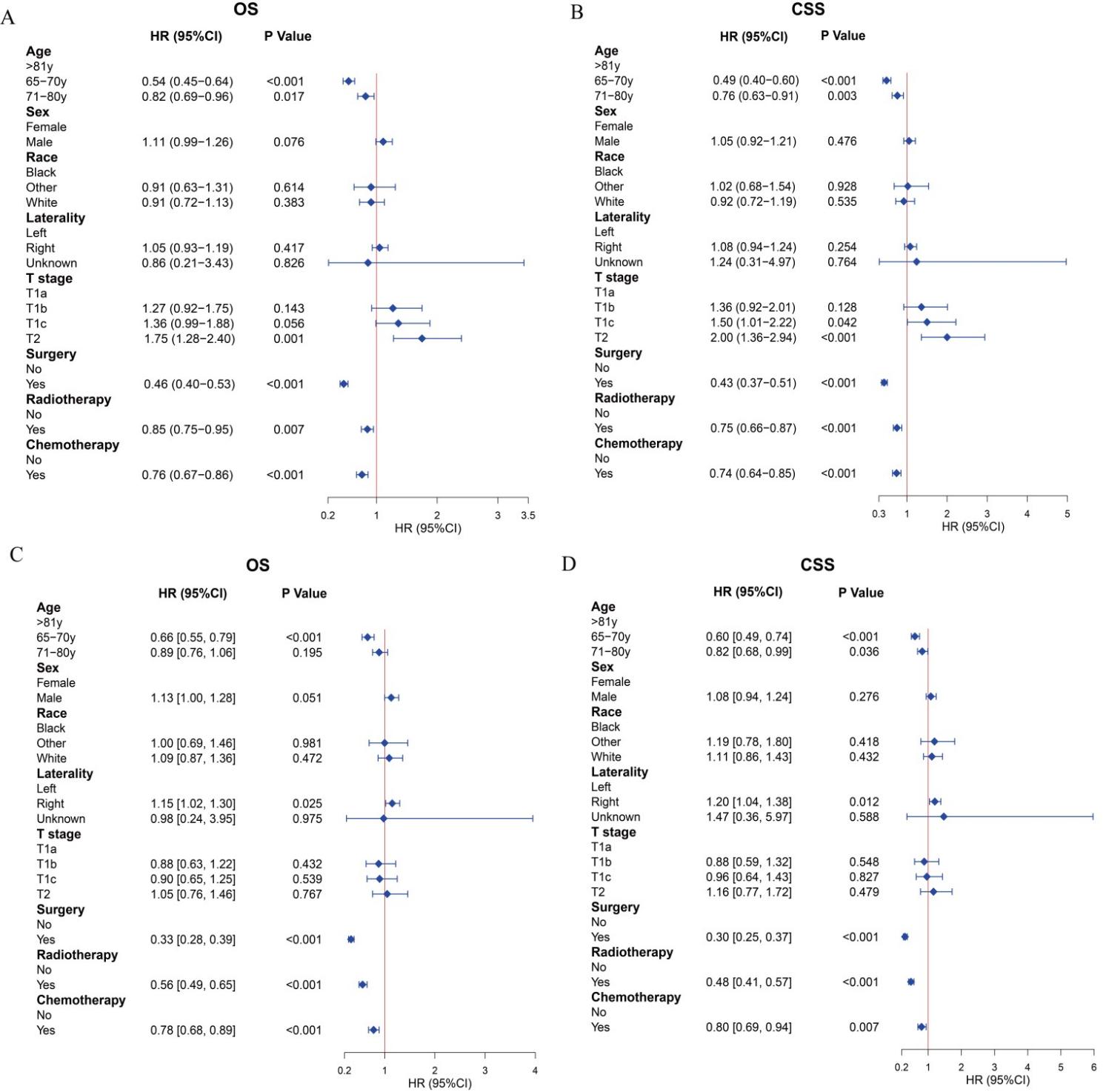


**Supplementary Figure 1.** Cox regression analysis for overall survival (OS)and cancer-specific survival(CSS) of older patients with T1-2N0M0 small cell lung cancer (SCLC)before propensity score matching. (A) Univariate Cox analysis for OS; (B) Univariate Cox analysis for CSS; (C) Multivariate Cox analysis for OS; (D) Multivariate Cox analysis for CSS.

**
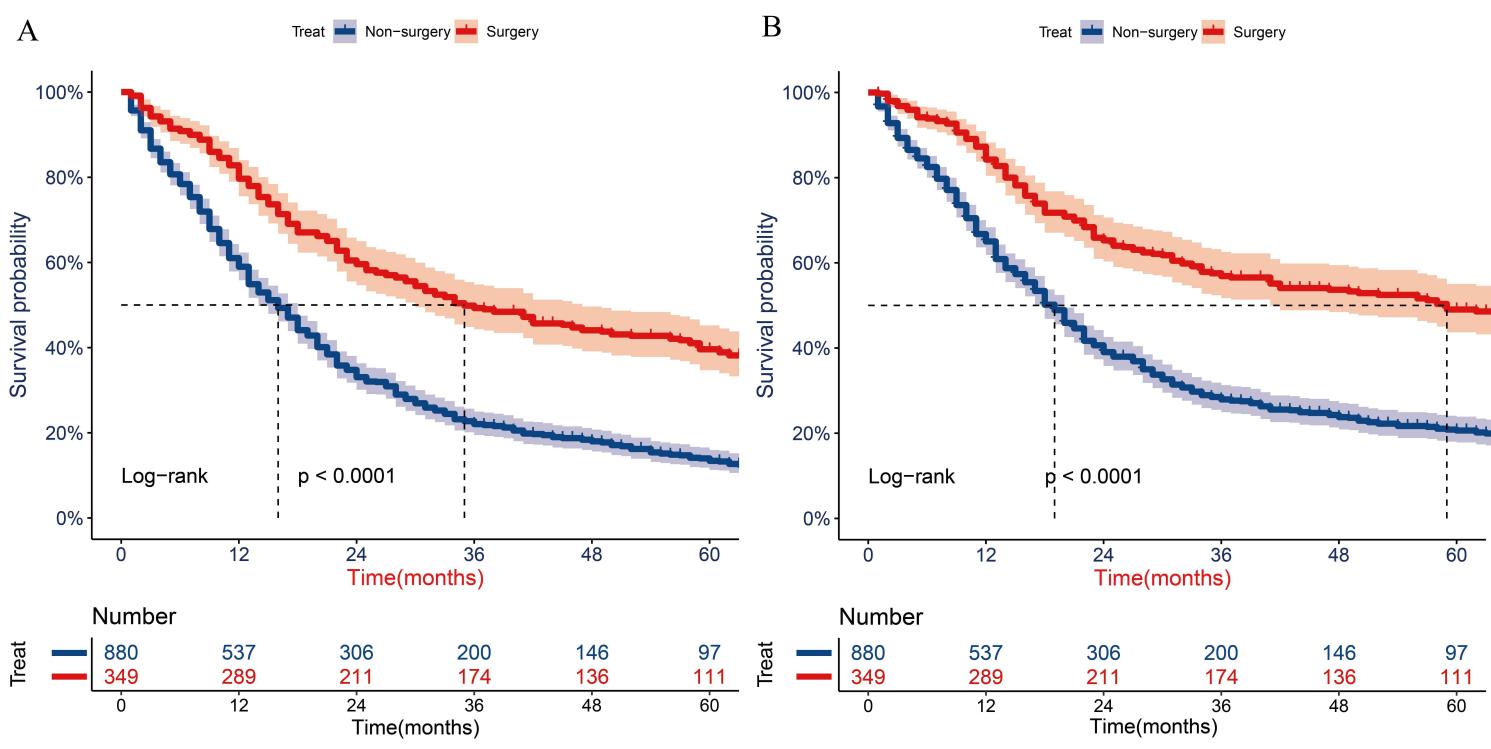
**

**Supplementary Figure 2.** Survival analysis for overall survival (OS) and cancer-specific survival (CSS) of older patients with T1-2N0M0 small cell lung cancer (SCLC) before propensity score matching. (A) KM curves of OS; (B) KM curves of CSS.
